# Immune heterogeneity of non-psychotic mental disorders

**DOI:** 10.1192/j.eurpsy.2021.1261

**Published:** 2021-08-13

**Authors:** S. Zozulya, L. Androsova, I. Otman, T. Vetlugina, V. Nikitina, T. Klyushnik

**Affiliations:** 1 Laboratory Of Neuroimmunology, Mental Health Research Centre, Moscow, Russian Federation; 2 Laboratory Of Clinical Psychoneuroimmunology And Neurobiology, Mental Health Research Institute, Tomsk National Research Medical Center, Tomsk, Russian Federation

**Keywords:** non-psychotic mental disorders, inflammatory markers

## Abstract

**Introduction:**

Current studies indicate the involvement of inflammation in the pathogenesis of chronic non-infectious diseases, and therefore it is of interest to study the role of inflammation markers in non-psychotic mental disorders (NPMD).

**Objectives:**

To identify a number of inflammatory markers in serum of patients with NPMD.

**Methods:**

73 patients with NPMD were examined (F43.2; F06.6). The comparison group consisted of 76 patients with endogenous psychosis (EGP) (F20.0; F25.0). The control group included 80 healthy people. The serum activity of leukocyte elastase (LE), α1-proteinase inhibitor (α1-PI) and the level of autoantibodies (aAb) to neuroantigens were determined.

**Results:**

Three groups of patients with different variants of inflammatory response to the pathological process were identified. In group 1 (23.3%), all indices corresponded to the control values, which indicated the absence of the pathological process in brain. In group 2, there was a significant increase in activity both LE and α1-PI compared to control (p<0.05). This type of immune reaction characterized a balanced inflammatory response. It was found in 52% of patients with NPMD and in all patients with EGP. The aAb level also exceeded the control values (p<0.05). Group 3 (24.7%) showed an increase in α1-PI activity (p<0.05), but not in LE activity compared to control. Insufficient LE activity reflects a decrease in the functional activity of neutrophils.

**Conclusions:**

The immune heterogeneity of NPMD according to the level of inflammatory markers was identified. 52% of patients with NPMD have a pronounced activation of inflammatory reactions accompanied by increased levels of aAb to neuroantigens.

